# Biomonitoring–Health Risk Nexus of Potentially Toxic Metals on *Cerithidea obtusa*: A Biomonitoring Study from Peninsular Malaysia

**DOI:** 10.3390/foods12081575

**Published:** 2023-04-07

**Authors:** Chee Kong Yap, Khalid Awadh Al-Mutairi

**Affiliations:** 1 Department of Biology, Faculty of Science, Universiti Putra Malaysia (UPM), Serdang 43400, Malaysia; 2 Department of Biology, Faculty of Science, University of Tabuk, Tabuk P.O. Box 741, Saudi Arabia; kmutairi@ut.edu.sa

**Keywords:** health risk, toxic metals, mangrove snails, consumption, biomonitoring

## Abstract

The present study aimed to assess the human health risks of six potentially toxic metals (PTMs) (Cd, Cu, Fe, Ni, Pb and Zn) in 21 populations of popular mangrove snails, *Cerithidea obtusa*, collected from Malaysia. In general, the concentrations (mg/kg wet weight) of Cd (0.03–2.32), Cu (11.4–35.2), Fe (40.9–759), Ni (0.40–6.14), Pb (0.90–13.4) and Zn (3.11–129) found in the snails in all populations were lower than the prescribed maximum permissible limits (MPL)s for Cd, Cu, Ni, Pb and Zn. However, in the investigated snail populations, Cd (14%), Pb (62%), Cu (19%), and Zn (10%) were found in exceedance of the MPL of the respective metal. The target hazard quotient (THQ) values in all populations for Cu, Ni, Fe and Zn were all found to be below 1.00. However, for the THQ values of Cd and Pb, two populations exceeded 1.00, while others were below the threshold level. The estimated weekly intake (EWI) of all six metals for all populations was only 0.03–4.65% of the provisional tolerable weekly intake. This conclusively indicates that, based on the EWI, there are no health risks of the six PTMs in the consumption of snails from Malaysia since the assessments are dependent on the consumers’ body weight and consumption rate. Nonetheless, the present results indicate that the amounts of snails consumed should be limited to minimize the potential health risks of PTMs to consumers. The relatively low and weak but positive correlations of Cu, Ni, Pb and Zn between *C. obtusa* and their habitat sediments indicate that *C. obtusa* can be a potential biomonitor for Cu, Ni, Pb and Zn. This is important for effective mangrove management from the perspective of the sustainable resources from the intertidal mangrove environment. Hence, the biomonitoring–health risk nexus of PTMs in mangrove snails is proposed in the present study.

## 1. Introduction

Global seafood consumers are increasingly concerned about potentially toxic metals and prefer safe, high-quality, and potentially toxic metal (PTM)-free products [1]. A significant impact of marine coastal pollution is metal-contaminated seafood, which has raised public attention due to its potential risks to human health [2]. Studies of PTMs in seafood have been increasing in the literature since the biomonitoring data are related to the human health risks of metal toxicity [1,2,3,4,5,6,7,8,9,10,11,12,13,14,15,16,17,18,19,20,21,22,23,24,25]. In particular, many such biomonitoring studies with health risk assessments were reported from Pakistan [1], Thailand [2], Brazil [3], Serbia [4], China [5,19,21,24], Nigeria [6,25], Kuwait [8], Malaysia [9,26], Vietnam [11], Bosnia and Herzegovina [12], Romania [15], Serbia [16], Hungary [20] and Turkey [22]. The six PTMs that were investigated in the present study were Cd, Cu, Fe, Ni, Pb and Zn because they are common anthropogenic metals and could potentially cause human health risks [1,2,3,4,5,6,7,8,9,10,11,12,13,14,15,16,17,18,19,20,21,22]. All the studies reported [1,2,3,4,5,6,7,8,9,10,11,12,13,14,15,16,17,18,19,20,21,22,23,24,25,26,27,28,29,30] did indicate that the ultimate rationale for using the molluscs when biomonitoring PTMs in coastal waters is that they are consumed extensively in some areas of the world and hence pose a risk to human health [26]. Thus, this was followed by human health risk assessments of the monitored PTMs in seafood [27,28,29,30].

Ni is present in all soil types, meteorites and volcanic eruptions. Municipal and industrial waste, the use of liquid and solid fuels and industry all potentially contaminate the environment with Ni. Most frequently, oxygen or sulphur are combined with nickel in the atmosphere to produce oxides or sulphides in the Earth’s crust [31,32] There is no doubt that occupational exposure to Ni or the widespread use of Ni in a number of industries negatively affects human health. Many goods use Ni metal and its derivatives, including batteries, alloys, and stainless steel [33]. Pb is a non-essential element that has been linked to nephrotoxicity and neurotoxicity, among other health issues [34]. This dangerous metal may seriously threaten public health since it may build up to large quantities in the human body and is not known to play any biological function [35]. Pb is carried by the body’s mineralising processes and soft tissues, including the blood, liver, kidneys, and bones. Pb poisoning can cause children to have lower IQs and less intellectual growth time [36]. In the non-smoking population, ingestible food is the main source of Cd consumption. As it accumulates in a person’s body, it can cause cancer, bone damage, renal dysfunction and other issues [37].

Cu is a necessary component of various enzymes that are essential to all living things. It is required for the production of haemoglobin [38]. Marine molluscs are a key source of Cu for human health because their blood includes hemocyanin, a respiratory protein that generally contains Cu in molluscs [38]. Additionally, Fe is a trace element that is essential for human nutrition and supports essential body functions in the form of haemoglobin, which transports oxygen in the blood [39]. Fe typically is not regarded as harmful to health unless it is ingested at very high doses. Accidental or intentional overdose resulting in acute iron overload can be lethal. Persistent iron overload may eventually impair important organs, including the liver and heart [39]. Lastly, Zn is also essential for human nutrition [40]. Given that it is a necessary component of cells and is an enzyme cofactor, it is one of the most important trace elements for metabolic processes in the human body [41]. Although it is an essential element for human health, excessive dietary Zn consumption can cause poisoning symptoms, such as nausea and fever, and major health problems, such as protein metabolism problems and pancreatic damage [41,42]. According to some experts, chronic exposure to Cu and Zn may induce Parkinson’s disease [38].

The mangrove snail *Cerithidea obtusa* (family: Potamididae), which is locally known as “siput sedut” or “siput Belitung” in Malaysia, has been priced at an average of MYR 15.36/kg, and the production (landing) of this snail species was reported at 42 tonnes in 2021 [43]. Compared to the popular mussel *Perna viridis*, which has been priced at an average of MYR 8.89/kg, the production (landing) of this mussel species was reported at 78 tonnes in 2021 [43]. This shows that although the production of *C. obtusa* was about two times lower than *P. viridis*, the price of the snails was about two times higher than that of the mussels. This indicates that *C. obtusa* is an increasingly high-priced seafood, at least in Malaysia. Most of the snails are sold locally and are highly consumed by Malaysians since the snails can be found in most local markets. Studies on potentially toxic metals in the mangrove snails have been reported in the literature [44,45,46,47,48,49,50,51,52,53,54,55,56,57,58,59,60,61,62,63]. In particular, biomonitoring using *C. obtusa* was reported from Indonesia [31,43,45,49], Malaysia [46,47,49,50,59,60], Vietnam [57] and India [48,52,54]. However, there has been no discussion on the connection between the water–energy–seafood nexus and PTM biomonitoring in the edible mangrove snails.

The aims of this study were to determine the levels of six PTMs (Cu, Cd, Fe, Ni, Pb and Zn) in the total soft tissues of *C. obtusa* snails from Peninsular Malaysia and to evaluate the public health risks associated with the consumption of the mangrove snails. Later, the biomonitoring of PTMs in the snails will be discussed from the perspective of the water–energy–seafood nexus.

## 2. Materials and Methods

### 2.1. Samplings

**Figure 1:**
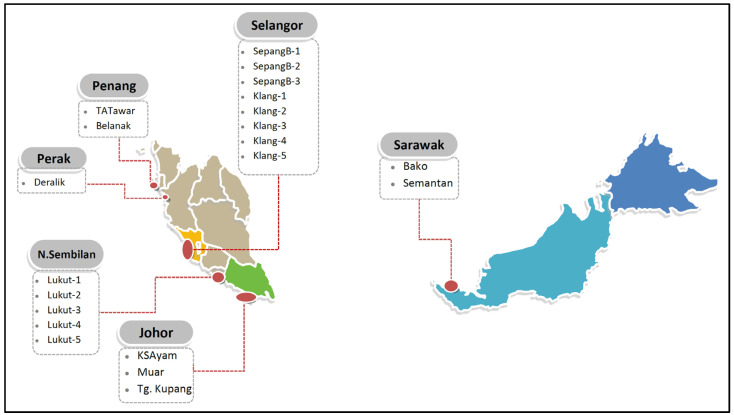
Sampling sites of the present study at the mangrove areas of Malaysia. Map has been modified from yourfreetemplate.com.

Nineteen populations of *C. obtusa* snails and their habitat surface sediments (1–10 cm) were collected from sampling sites in the mangrove on the west coast of Peninsular Malaysia, and two populations (Bako and Semantan) were purchased from Sarawak (Figure 1). The sampling descriptions are provided in Table S1.

### 2.2. Sample Preparation and Metal Analysis

Approximately 15–20 snails were collected from each sampling site. In the laboratory, the snails were measured for their shell lengths. The soft tissues were dissected from the shells and pooled. All the pooled total soft tissue samples were dried at 100 °C in an oven for three days until a constant dry weight was achieved. The oven-dried total soft tissues of the snails were digested in concentrated nitric acid (AnalaR grade; BDH 69%), while the oven-dried and 63 µm sieved sediments were digested in a combination of concentrated nitric acid (HNO_3_; AnalaR grade; BDH 69%) and concentrated perchloric acid (HClO_4_; AnalaR grade; BDH 60%) in a ratio of 4:1. They were placed in a hot-block digestor for one initial hour at a low temperature (40 °C). After that, the temperature was raised to 140 °C for three hours [64]. After being digested, the samples were diluted in 40 mL of double-distilled water. The samples were then filtered using Whatman No. 1 filter paper, and the filtrates were kept until metal determination in pill boxes that had been acid-washed. Using a PerkinElmer Model AAnalyst 800 air-acetylene flame atomic absorption spectrophotometer, the prepared samples were determined for Cd, Cu, Fe, Ni, Pb and Zn.

### 2.3. Quality Monitoring and Assurance

All glassware and equipment utilised were acid-cleaned to prevent contamination, and the accuracy of the analysis was verified using blanks. For the purpose of validating the data, certified values of the Certified Reference Material for mussel tissue (NIST 2976) and dogfish liver (DOLT-3, National Research Council Canada) were compared to the recoveries for each, as shown in Table S2.

### 2.4. Human Health Risk Assessments

The PTM data on a dry weight (dw) basis were transformed into a wet weight (ww) basis for the human health risk assessment using a conversion factor of 0.24 for *C. obtusa* [unpublished data]. Three evaluations were conducted to determine the health risk assessment resulting from the consumption of the snails.

#### 2.4.1. Direct Comparisons with Seafood Safety Guidelines

Only the maximum permissible limit (MPL) of Ni, also known as the action level (80 mg/kg WW) for molluscan shellfish, was included in the comparison [65]. The comparison for Cd was based on the Cd MPL (1.00 mg/kg ww) established by Malaysian Food Regulations (MFR) [66], the Commission Regulation of the European Union [67], the 2.00 mg/kg ww set by the Codex Alimentarius Commission in marine bivalves [68] and the MPLs (1–2 mg/kg ww) of the FAO compilation of the legal limits by FAO (which are equivalent to the MPL) of Cd in fish/fish products/shellfish.

For Pb, the comparison was based on the Pb MPLs suggested by the commission regulation of the European Union (1.50 mg/kg ww EC [67]), US Food and Drug Administration (1.70 mg/kg ww USFDA/CFSAN [65]), ANZFA (2.00 mg/kg ww [69]), Malaysian Food Regulations 1985 (2.00 mg/kg ww [66]) and the legal limits of Pb (2–10 mg/kg ww) compiled by the FAO [70], based on the limits from New Zealand, the UK and Australia.

The comparisons for Cu were based on the Cu MPLs recommended by the Malaysian Food Regulations (30 mg/kg ww [66]) and the FAO [70], which were based on the nations of New Zealand, the UK and Australia, with the range of the legal limit of Cu set as 20–70 mg/kg ww. The comparisons for Zn were based on the Malaysian Food Regulations (100 mg/kg ww [66]) and the MPLs recommended by the FAO [70], which ranged from 40 to 150 mg/kg ww based on the MPLs from New Zealand, the UK and Australia. Nevertheless, before 2015, there were scarcely any Fe upper limits established by the WHO, FDA, FAO or other nations. Previously, the JECFA [39], based on the opinions of a global panel of experts, concluded that uncertainty remained about the maximum level of Fe that can be tolerated. Thus, it is impossible to compare MPLs to the current Fe data.

#### 2.4.2. Target Hazard Quotient

The estimated daily intake (EDI) must first be computed before determining the target hazard quotient (THQ). The EDI calculates the specific metal intake by employing body weight (bw) and the rate of snail consumption. It ascertained out according to Equation (1):

EDI = (Mc × CR)/bw(1)

where CR represents the average daily consumption rates (40 g/person/day) of category molluscs for Malaysian adults, based on 2675 respondents (Malay: 76.9%; Chinese: 14.7%; Indian: 8.4%), and Mc represents the metal content in the samples (mg/kg) on a WW basis [71]. According to Nurul Izzah et al. [71], the typical adult population of Malaysia has a bw of 62 kg, and the high-level consumer rate is expected to be twice that amount.

The THQ was later determined using Equation (2):

THQ = EDI/ORD(2)

where ORD represents the oral reference dose.

The oral reference dose (ORD) estimates a contaminant’s lifetime daily consumption that is unlikely to result in adverse health effects [72]. The ORD values (µg/kg/day) used in this study were: Cd: 1.00; Cu: 40.0; Ni: 20.0; Fe: 700; Pb: 3.50; Zn: 300, provided by the USEPA’s regional screening level [72].

#### 2.4.3. Comparisons between Estimated Weekly Intake (EWI) and Provisional Tolerable Weekly Intake (PTWI)

The provisional tolerated weekly intake (PTWI), was developed by the Joint FAO/WHO Expert Committee on Food Additives [73]. The risk of food consumption to human health was evaluated by calculating weekly metal exposures and comparing the findings to the pertinently advised PTWI values. The PTWI, measured in mg/kg of bw, is the amount of a material that is believed to be present in food or drinking water which may be taken without causing substantial harm to health over the course of a lifetime [74]. The amount of seafood from this research that exceeded the PTWI limits was calculated as a consequence.

All the values of the oral reference dose (ORD, µg/kg bw/day) and PTWI (mg/kg bw/week) for Cd, Cu, Ni, Fe, Pb and Pb and Zn used in the present study are presented in Table S3. The PTWI values for Zn were recalculated from the provisional tolerable daily intake (PTDI), based on JECFA [40,75]. The PTWI values for Fe were recalculated from provisional maximum tolerable daily intake (PMTDI), based on JECFA [39,75]. The PTWI values for Cu were recalculated from PMTDI, based on JECFA [40,75]. The PTWI values for Ni were recalculated from the tolerable daily intake (TDI), based on EFSA [76]. The PTWI values for Pb were recalculated from TDI, based on JECFA [77]. The PTWI values for Cd were recalculated from provisional tolerable monthly intake (PTMI), based on JECFA [75,77]. As a result, 5642 µg/week of Ni is required to maintain an adult’s average weight of 62 kg in Malaysia; 1302 µg/week of Pb is required to maintain an adult’s average weight of 62 kg in Malaysia; 361.5 µg/week of Cd is required to maintain an adult’s average weight of 62 kg in Malaysia; 217,000 µg/week of Cu is required to maintain an adult’s average weight of 62 kg in Malaysia; 347,200 µg/week of Fe is required to maintain an adult’s average weight of 62 kg in Malaysia; and 434,000 µg/week of Zn is required to maintain an adult’s average weight of 62 kg in Malaysia.

The value of the estimated weekly intake (EWI) for each metal in *C. obtusa* was calculated in Equation (3), as follows, to determine the risk of exposure from consumption:

EWI = EDI × 7(3)

where EDI represents the estimated daily intake calculated in Equation (1) and multiplied by seven because there are 7 days in a week.

### 2.5. Statistics Analysis

The overall data statistics in the present study were generated using the KaleidaGraph (Version 3.08, Sygnergy Software, Eden Prairie, MN, USA). All graphical bar charts were also created. A linear regression for the graphs was used to model the correlation of the metal levels between the snails and habitat sediments [78].

## 3. Results

In Table 1, the mean concentrations and their overall statistics are listed. For a valid comparison of metal concentrations with MPLs for food safety standards published in the ww basis, all metal levels in the dw basis were converted into the ww basis by applying a conversion factor of 0.24.

### 3.1. Comparison with Food Safety Guidelines of Potentially Toxic Metals

**Figure 2:**
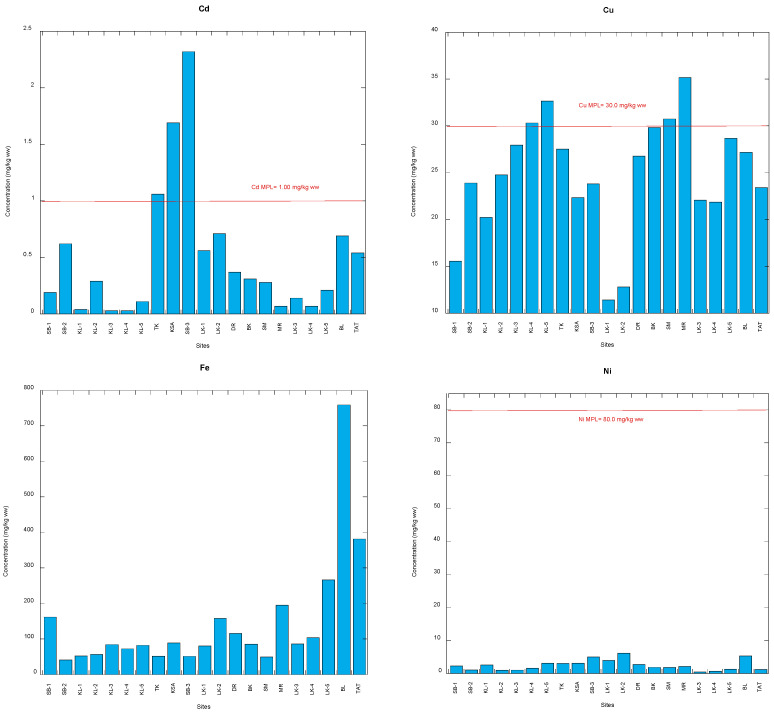

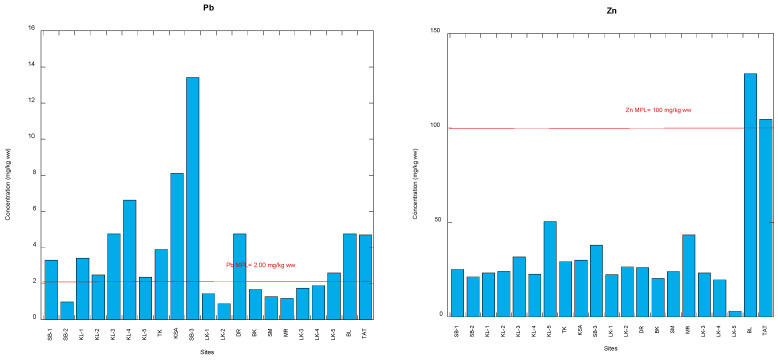
Comparisons of six potentially toxic metals concentrations (mg/kg wet weight (ww)) between the total soft tissues of 21 populations of *Cerithidea obtusa* and their maximum permissible limits (MPL). Note: SB = Sepang Besar; KL = Klang; TK = Tanjung Kupang; KSA = Kampung Sg. Ayam; LK = Lukut; DR = Deralik; BK = Bako; SM = Sematan; MR = Muar; BL = Belanak; TAT = Teluk Ayer Tawar (Table S1).

The concentrations of Ni in *C. obtusa* ranged from 1.67 to 25.6 mg/kg dry weight (0.40–6.14 wet weight) in the 21 snail populations (Table 1; Table S4). MPLs for Ni are not commonly found in the literature [26]. However, the US Food and Drug Administration [65] established the only Ni MPL that is currently accessible, known as the action level (Ni: 80 mg/kg ww). As a result, the Ni levels in all *C. obtusa* populations were significantly below the Ni action limit (Figure 2). Therefore, there is no non-carcinogenic risk of Ni in snail consumption from the present study.

The concentrations of Cd in *C. obtusa* ranged from 0.11 to 9.65 mg/kg dry weight (0.03–2.32 wet weight) in the 21 snail populations (Table 1 and Table S4). There were three populations (TK, KSA and SB-3) recorded to have a higher concentration of Cd than the Malaysian MPL (1.00 m mg/kg ww [66]). The other 18 populations (86%) were found to be below the Cd MPL (Figure 2). When compared to the Cd legal limits (1.00–2.00 mg/kg ww) established by the FAO [70] in fish/fish products/shellfish, the 2.00 mg/kg ww set by the Codex Alimentarius Commission in marine bivalves, [68], and the MPL (2.00 mg/kg ww) set by ANZFA [69], a similar observation can be made. However, all populations were below the MPL (4.00 mg/kg ww) set by the USFDA/CFSAN [65]. However, public concern regarding Cd toxicity remains such that the three populations, TK, KSA and SB-3, require extra attention.

The concentrations of Pb in *C. obtusa* ranged from 3.75 to 55.9 mg/kg dry weight (0.90–13.4 wet weight) in the 21 snail populations (Table 1 and Table S4). There were 13 populations (62%) that were recorded to have a higher concentration of Pb than the Malaysian MPL (2.00 mg/kg ww [66]). The other eight populations (38%) were found to be below the Pb MPL (Figure 2). Almost similar conclusions can be reached when the Pb safety guidelines are compared to those suggested by the commission regulation of the European Union (1.50 mg/kg ww [67]), the US Food and Drug Administration (1.70 mg/kg ww [65]), and ANZFA (2.00 mg/kg ww [69]). When comparing the Pb legal limits (2.00–10.0 mg/kg ww) established by the FAO [70], only the SB-1 population exceeded the highest Pb legal limit (10.0 mg/kg ww) (Figure 2). However, the 62% of the population that exceeded the MPL require further caution from the public perspective.

The concentrations of Cu in *C. obtusa* ranged from 47.6 to 147 mg/kg dry weight (11.4–35.2 wet weight) in the 21 snail populations (Table 1 and Table S4). There were four populations (KL-4, KL5, SM and MR) that were recorded to have a higher concentration of Cu than the Malaysian MPL (30.0 mg/kg ww [66]) The other 17 populations (81%) were found to be below the Cu MPL (Figure 2). When comparing the Cu legal limits (20–70 mg/kg ww) established by the FAO [70], all populations were found to be well below the highest Cu legal limit (70 mg/kg ww). However, the four populations require public concern with respect to the consumption of the snails.

The concentrations of Fe in *C. obtusa* ranged from 171 to 3162 mg/kg dry weight (40.9–759 wet weight) in the 21 snail populations (Table 1 and Table S4). Until to 2015, there were scarcely any Fe maximum limits established by the WHO, FDA, FAO or other nations. Prior to the present, the JECFA [39], based on the opinions of a global panel of experts, concluded that there some doubt remained regarding the maximum tolerable level of Fe. As a result, it is impossible to compare the MPLs with the available Fe data. However, there were hardly any Fe maximum limits set by the WHO/FDA/FAO or other countries until 2015. Earlier, based on the collective views of an international group of experts, the JECFA [39] concluded that there uncertainty remained regarding the maximum tolerable level of Fe. Therefore, comparing MPLs with the present Fe data is impossible.

The concentrations of Zn in *C. obtusa* ranged from 12.9 to 536 mg/kg dry weight (3.11–129 wet weight) in the 21 snail populations (Table 1 and Table S4). There were two populations (BL and TAT) that were recorded to have a higher concentration of Zn than the Malaysian MPL (100 mg/kg ww [66]). The other 19 populations (90%) were found to be below the Zn MPL (Figure 2). When comparing the Zn legal limits (40–150 mg/kg ww) established by the FAO [70], all populations were found to be below the highest Zn legal limit (150 mg/kg ww). Hence, the present Zn levels in *C. obtusa* were all well below the Zn safety guidelines except for the two populations, BL and TAT. Therefore, snail consumption from BL and TAT could pose a Zn risk to human health.

### 3.2. Target Hazard Quotients

**Figure 3:**
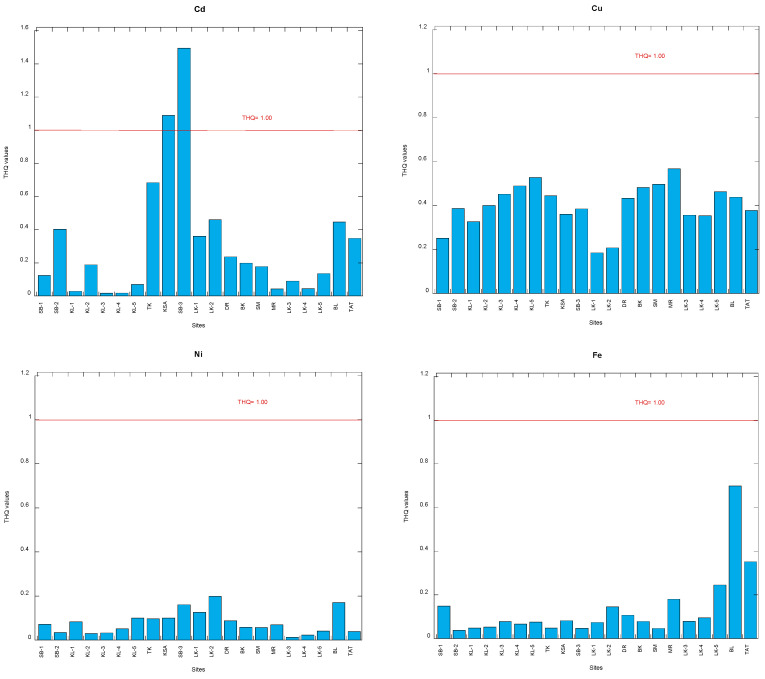

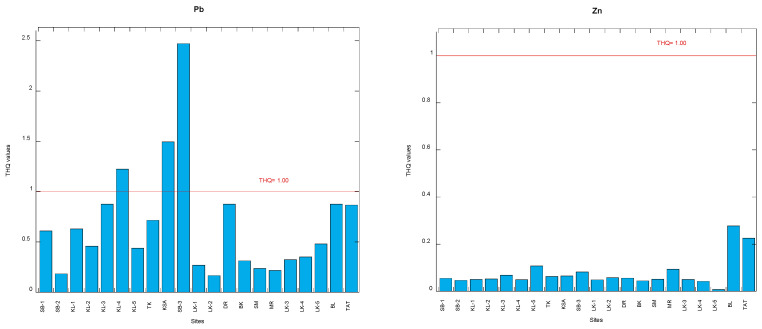
Comparison of values of the target hazard quotient (THQ) for six potentially toxic metals between the total soft tissues of 21 populations of *Cerithidea obtusa* and the THQ threshold of 1.00. Note: SB = Sepang Besar; KL = Klang; TK = Tanjung Kupang; KSA = Kampung Sg. Ayam; LK = Lukut; DR = Deralik; BK = Bako; SM = Sematan; MR = Muar; BL = Belanak; TAT = Teluk Ayer Tawar (Table S1).

The Ni EDI and Ni THQ values in the snails are summarized in Table 2 (Table S5). The Ni EDI (µg/kg body weight/day) and THQ (unitless) values ranged from 0.26 to 3.96 and from 0.013 to 0.198 in the 21 populations. Clearly, these Ni THQ values are significantly lower than 1.00 (Figure 3). Therefore, there is no non-carcinogenic risk of Ni in the consumption of snails from Malaysia.

The Cd EDI and Cd THQ values in the snails are summarized in Table 2 (Table S5). The Cd EDI (µg/kg body weight/day) and THQ (unitless) values ranged from 0.02 to 1.49 and from 0.02 to 1.49 in the 21 populations. Two populations (KSA and SB-3) clearly exceeded the Cd THQ of 1.00 (Figure 3). Therefore, there is no non-carcinogenic risk of Cd in consuming snails from Malaysia except for the two populations from KSA and SB-3.

The Pb EDI and Pb THQ values in the snails are summarized in Table 2 (Table S5). The Pb EDI (µg/kg body weight/day) and THQ (unitless) values ranged from 0.58 to 8.65 and from 0.16 to 2.47 in the 21 populations. Clearly, there were three populations (SB-3, KSA and KL-4) that exceeded the Pb THQ of 1.00, while others were lower than 1.00 (Figure 3). Therefore, there is no non-carcinogenic risk of Pb in consuming snails from Malaysia except for the three populations SB-3, KSA and KL-4.

The Cu EDI and Cu THQ values in the snails are summarized in Table 2 (Table S5). The Cu EDI (µg/kg body weight/day) and THQ (unitless) values ranged from 7.37 to 22.7 and from 0.18 to 0.57 in the 21 populations. Clearly, all the 21 populations had Cu THQ values which were significantly lower than 1.00 (Figure 3). Therefore, there is no non-carcinogenic risk of Cu in consuming snails from Malaysia.

The Fe EDI and Fe THQ values in the snails are summarized in Table 2 (Table S5). The Fe EDI (µg/kg body weight/day) and THQ (unitless) values ranged from 26.4 to 490 and from 0.04 to 0.70 in the 21 snail populations. Clearly, all the 21 populations had Fe THQ values which were significantly lower than 1.00 (Figure 3). Therefore, there is no non-carcinogenic risk of Fe in consuming snails from Malaysia.

The Zn EDI and Zn THQ values in the snails are summarized in Table 2 (Table S5). The Zn EDI (µg/kg body weight/day) and THQ (unitless) values ranged from 2.01 to 83.0 and from 0.01 to 0.28 in the 21 snail populations. Clearly, all the 21 snail populations had Zn THQ values which were significantly lower than 1.00 (Figure 3). Therefore, there is no non-carcinogenic risk of Zn in consuming snails from Malaysia.

### 3.3. Comparisons between Estimated Weekly Intake (EWI) and Provisional Tolerable Weekly Intake (PTWI)

**Figure 4:**
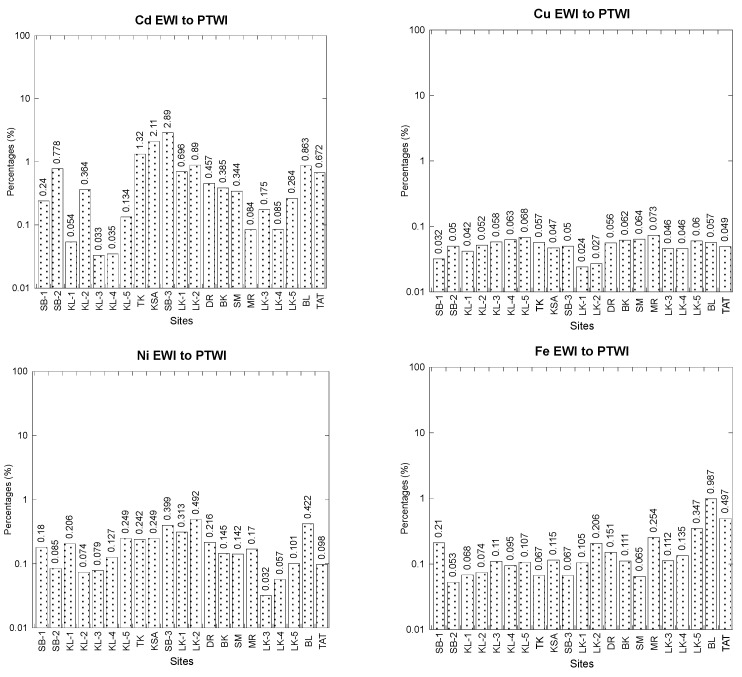

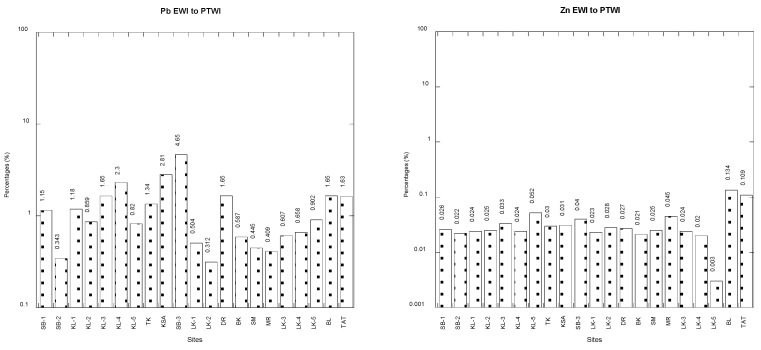
Comparison of values of estimated weekly intake (EWI, µg/kg body weight/week) and their percentages in comparison to provisional tolerable weekly intake (PTWI, µg/kg body weight/week) of six potential toxic metals in the total soft tissues of 21 populations of *Cerithidea obtusa* collected in the present study. Note: SB = Sepang Besar; KL = Klang; TK = Tanjung Kupang; KSA = Kampung Sg. Ayam; LK = Lukut; DR = Deralik; BK = Bako; SM = Sematan; MR = Muar; BL = Belanak; TAT = Teluk Ayer Tawar (Table S1).

The overall statistics of the EWI values and their percentages compared to the PTWI for Ni in the total soft tissues of 21 populations of *C. obtusa* are presented in Table 3 (Table S6). The Ni EWI values ranged from 1.81 to 27.7 µg/kg bw/week, which is only 0.032–0.492% of the Ni PTWI (Figure 4). Clearly, all the EWI values were significantly lower than the PTWI value (5642 µg/kg bw/week). Therefore, snail consumption was not considered to have adverse effects of Ni for consumers based on the FAO/WHO JECFA guidelines. The EFSA [76] set a tolerable daily intake (TDI) of 13.0 μg/kg bw. Hence, the PTWI of Ni is 91 µg/kg bw/week (13.0 μg/kg bw × 7 days). Subsequently, the Ni PTWI for an average adult in Malaysia, with a bw of 62 kg, is equivalent to 5642 µg/week.

The overall statistics of the EWI values EWI and their percentages compared to the PTWI for Cd in the total soft tissues of 21 populations of *C. obtusa* are presented in Table 3 (Table S6). The Cd EWI values ranged from 0.12 to 10.5 µg/kg bw/week, which is only 0.033–2.89% of the Cd PTWI (Figure 4). Clearly, all the EWI values were significantly lower than the Cd PTWI value (361.5 µg/kg bw/week). Therefore, snail consumption was not considered to have adverse effects of Cd for consumers based on the FAO/WHO JECFA guidelines. The total or average intake should be evaluated over months, and acceptable consumption should be evaluated over at least one month to evaluate the long- or short-term health hazards associated with Cd exposure [77]. To support this viewpoint, the Committee described the acceptable intake as a monthly figure in the form of a PTMI. The PTDI (g/kg bw/day) of Cd was determined using a provisional tolerated monthly intake (PTMI) of 25.0 g/kg bw, based on a month of 30 days [75,77]. Therefore, the provisional daily intake (PTDI) is equal to (25 g/kg bw/month)/(30 days) = 0.833 g/kg bw/day. Therefore, PTWI = (0.833 g/kg bw/day) × 7 days = 5.83 g/kg bw/week. A Malaysian adult weighing 62 kg has a Cd PTWI of 361.5 g per week, which is the equivalent.

Based on daily rice consumption, Horiguchi et al. [79] concluded that compared to other groups with lower Cd exposure, female Japanese farmers who had eaten foods containing Cd at a level near the current PTWI did not exhibit an increased development of renal tubular dysfunction. As Cd has spread throughout the environment due to industrial and agricultural activities, the effect of Cd pollution on public health is a serious issue. According to Oberdorster [80], the main way people are exposed to environmental Cd is by breathing in air containing Cd. However, another crucial exposure is the consumption of Cd-contaminated food, such as shellfish. Horiguchi et al. [81] investigated Cd exposure, accumulation, renal effects and the association between age and Cd effects in female farmers who resided in Cd-polluted regions. They concluded that consuming rice contributed to excessive Cd accumulation and caused renal function in older women to decline in polluted environments. In Japan’s Jinzu River Basin, which has high levels of Cd contamination that is linked to osteomalacia and renal anaemia, Itai-Itai disease, the most severe form of chronic Cd poisoning, first appeared among female farmers [82]. The quantity of mussels that may be ingested without exceeding the JECFA limit for Cd is the highest in Croatia (0.57 kg per week; Cd mean: 0.61 mg/kg ww), where the highest Cd mean concentrations were found, and the lowest in Albania (0.08 mg/kg ww), according to research by Jovic and Stankovic [35]. Comparatively, Jovic and Stankovic [35] found that the predicted weekly Cd intake for high mollusc consumers was 0.076 mg Cd/person/week and 0.15 mg Cd/person/week, based on the measured Cd concentrations in mussels from the Adriatic Sea.

The overall statistics of the EWI values and their percentages compared to the PTWI for Pb in the total soft tissues of 21 populations of *C. obtusa* are presented in Table 3 (Table S6). The Pb EWI values ranged from 4.06 to 60.6 µg/kg bw/week, which is only 0.31–4.65% of the Pb PTWI (Figure 4). Clearly, all the EWI values were significantly lower than the Pb PTWI value (1302 µg/kg bw/week). Therefore, snail consumption was not considered to have adverse effects of Pb on consumers, based on the FAO/WHO JECFA guidelines. JECFA [35] stated that the upper end of the range (3 g/kg bw/day) was used to compute the TDI (g/kg bw/day) of Pb for adults. The PTWI equals (3.00 g/kg bw/day) × 7 days (21.0 g/kg bw/week). Thus, 1302 g/week of Pb equals a 62 kg adult’s average bw in Malaysia. The nations with the greatest Pb mean content (1.01 mg/kg ww) are Italy and Slovenia, with the lowest amount of mussels that can be consumed each week (0.26 kg) and the largest amount (0.78 kg; Pb mean: 0.34 mg/kg ww), respectively. Comparatively, Jovic and Stankovic [35] discovered that based on the measured Pb contents in mussels from the Adriatic Sea, the estimated weekly intake of Pb for high mollusc consumers was 0.13 mg/person/week and 0.25 mg/person/week.

The overall statistics of the EWI values and their percentages compared to the PTWI for Cu in the total soft tissues of 21 populations of *C. obtusa* are presented in Table 3 (Table S6). The Cu EWI values ranged from 51.5 to 159 µg/kg bw/week, which is only 0.024–0.073% of the Cu PTWI (Figure 4). Clearly, all the EWI values were significantly lower than the Cu PTWI value (217,000 µg/kg bw/week). Therefore, snail consumption was not considered to have adverse effects of Cu to consumers, based on the FAO/WHO JECFA guidelines. The PTDI (µg/kg bw/day) of Cu was calculated from a provisional maximum tolerable daily intake (PMTDI) of 0.50 mg/kg bw/day [40,75]. Therefore, the Cu PTWI = (0.50 mg/kg bw/day) × 7 days = 3.50 mg/kg bw/week. Subsequently, the Cu PTWI for an average adult in Malaysia with a bw of 62 kg is equivalent to 217 mg/week (217,000 µg/week). According to the Institute of Medicine [83], the tolerable upper intake levels for Cu (upper limit for elemental Cu) were proposed as 10,000 mg/day (or mg/kg bw/day). According to a study by Jovic and Stankovic [35], the highest limit (140 kg per week) of mussels that may be consumed without harm is found in Montenegro, whereas Croatia has the lowest Cu mean level (4.46 mg/kg ww). In contrast, Jovic and Stankovic [35] discovered that the weekly intake of Cu was projected to vary from 0.19 to 0.56 mg/person/week for high mollusc users and 1.12 mg/person/week based on the mean Cu concentrations in mussels from the Adriatic Sea.

The overall statistics of the EWI values and their percentages compared to the PTWI for Fe in the total soft tissues of 21 populations of *C. obtusa* are presented in Table 3 (Table S6). The Fe EWI values ranged from 185 to 3427 µg/kg bw/week, which is only 0.053–0.987% of the Fe PTWI (Figure 4). Clearly, all the EWI values were significantly lower than the Fe PTWI value (347,200 µg/kg bw/week). Therefore, snail consumption was not considered to have adverse effects of Fe on consumers, based on the FAO/WHO JECFA guidelines. The PTDI (mg/kg bw/day) of Fe was calculated from a provisional maximum tolerable daily intake (PMTDI) of 0.80 mg/kg bw/day for all sources except for Fe oxide colouring agents, supplemental Fe for pregnancy and lactation and supplemental iron for specific clinical requirements) [39,75]. Therefore, the Fe PTWI = (0.80 mg/kg bw/day) × 7 days = 5.60 mg/kg bw/week. Subsequently, the Fe PTWI for an average adult in Malaysia with a bw of 62 kg is equivalent to 347.2 mg/week (347,200 µg/week). According to the Institute of Medicine [83], the tolerable upper intake levels for Fe (upper limit for elemental Fe) was proposed as 45 mg/day (or mg/kg bw/day). In a study by Jovic and Stankovic [35], it was shown that Albania had the lowest weekly intake of mussels (2.52 kg; Fe mean: 133.5 mg/kg ww) and Croatia had the highest (10.5 kg; Fe mean: 32.1 mg/kg ww). These percentages do not create much public worry, even though other meals may add to the PTWI of Fe because Fe is a vital component of living tissues. Jovic and Stankovic [35] estimated that high-level mollusc consumers (0.250 kg) would consume 33.4 mg Fe per week.

The overall statistics of the EWI values and their percentages compared to the PTWI for Zn in the total soft tissues of 21 populations of *C. obtusa* are presented in Table 3 (Table S6). The Zn EWI values ranged from 14.1 to 581 µg/kg bw/week, which is only 0.003–0.134% of the Zn PTWI (434,000 µg/kg bw/week) (Figure 4). Clearly, all the EWI values were significantly lower than the Zn PTWI value (434,000 µg/week). Therefore, snail consumption was not considered to have adverse effects of Zn on consumers, based on the FAO/WHO JECFA guidelines. The PTDI (mg/kg bw/day) of Zn was calculated from a provisional maximum tolerable daily intake (PMTDI) of 1.00 mg/kg bw/day [40,75]. Therefore, the Zn PTWI = (1.00 mg/kg bw/day) × 7 days = 7.00 mg/kg bw/week. Subsequently, the Zn PTWI for an average adult in Malaysia with a bw of 62 kg is equivalent to 434 mg/week (434,000 µg/week). According to the Institute of Medicine [83], the tolerable upper intake level for Zn (the upper limit for elemental Zn) was proposed as 40 mg/day (or mg/kg bw/day). According to Jovic and Stankovic [35], the lowest quantity of mussels (6.68 kg per week) with the highest Zn mean content (62.9 mg/kg ww) may be consumed in Croatia, and the highest amount (19.8 kg per week; Zn mean: 21.2 mg/kg ww) from Italy. In contrast, Jovic and Stankovic [35] discovered that the predicted weekly intakes of Zn for high-level mollusc consumers (7.86 mg/person/week) and 15.7 mg/person/week based on the calculated Zn concentrations in mussels from the Adriatic Sea.

### 3.4. Relationships of Metals between Snails and Their Habitat Sediments

**Figure 5:**
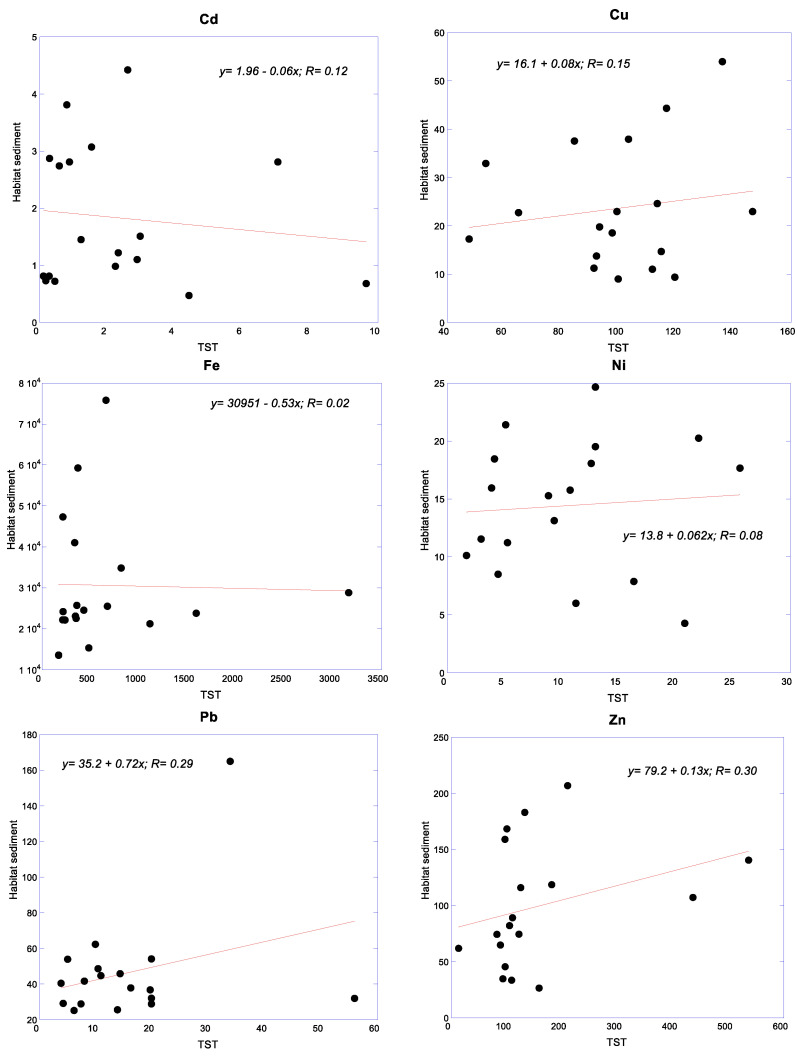
Relationships of metals between snails and their habitat sediments. All linear equations are based on N = 18. Note: TST = total soft tissues of *Cerithidea obtusa*.

The relationships of metals between snails and their habitat sediments are presented in Figure 5. There are positive relationships of Cu (0.08), Ni (0.062), Pb (0.72) and Zn (0.13) between the snails and their habitat sediments. Although the correlation coefficients (R values = 0.08–0.30) are relatively low and weak, these positive correlations indicate the potential of *C. obtusa* to be employed as a good biomonitor for Cu, Ni, Pb and Zn. On the other hand, there are negative or hardly any Cd (−0.06) and Fe (−0.53) relationships between the snails and their habitat sediments.

## 4. Discussion

### 4.1. General Low Health Risks with a Localized Elevation of Potentially Toxic Metals

In all snail populations, the concentrations of the six PTMs were generally lower than the prescribed MPLs for Cd, Cu, Ni, Pb and Zn. However, in all the snail populations investigated, Cd (14%), Pb (62%), Cu (19%) and Zn (10%) were found in exceedance of the MPL of the respective metal. The THQ values in all Cu, Ni, Fe and Zn in all snail populations were found below 1.00. However, for the THQ values of Cd and Pb, there were two and three populations for Cd and Pb, respectively, that exceeded 1.00, while the others were below the threshold level. This indicated a localized elevation of PTMs in the snail populations. However, it is comforting to note that the EWI values of all six metals for all populations were only 0.03–4.65% of the PTWI values for all metals studied, which indicates that the overall risk of the six metals in snail consumption is far from being a health risk concern. In other words, the comparison of values between the present EWI (EDI × 7 days) and the PTWI for all metals indicated for all six metals were far lower than the recommended PTWI values of the six metals. However, the EWI values of the metals are dependent on their consumption rate. The more consumed and the higher the frequency of consumption, the higher the EWI value is expected to be. Consuming snails over an extended period of time at these levels may increase the PTM load on the consumers. Hence, these EWI values could reach or exceed the PTWI values of all metals investigated. It should also be noted that the sources of metals can include all sources of meals such as fish, vegetables, meats, drinking water and other ingestible food items, all of which are potential sources of food items that can increase the EWI values.

It is rationally acceptable that the EWI values (which are based on the EDI) of the PTMs are considered the best health risk assessment, followed by the THQ values and lastly the MPL values. The MPL values are the last choice for the human health risk assessment since the factors of the snail consumption rate and the consumer body weight (related to age) are not taken into account. Between EWI and THQ values, the THQ values take lower confidence over the EWI values because the THQ values never consider the frequency of consumption per week, which is a more practical consumption behavioural habit of seafood consumers. Therefore, with the inclusion of the frequency of consumption rate per week and the body weight of consumers, the EWI is unarguably the best human health risk assessment of PTMs for seafood consumers. In addition, the use of EWI (in comparison to PTWI) values in the human health risk assessment of PTMs is well-reported in several studies [14,20,24,39,40].

Hence, the current study verified that the seafood *C. obtuse*, which is collected from Malaysian mangroves, is safe for human consumption. In order to provide more information and support the protection of human health, it is advised that more studies and evaluations of seafood quality be conducted [18]. In order to create appropriate and scientific rules on this region’s ecological and food safety, the government can utilize these facts as a trustworthy reference [19].

### 4.2. Biomonitoring of Potentially Toxic Metals Using Cerithidea obtusa for Effective Mangrove Ecosystem Management

The relatively low and weak but positive correlations of Cu, Ni, Pb and Zn between *C. obtusa* and their habitat sediments indicated that *C. obtusa* could be a potential biomonitor for Cu, Ni, Pb and Zn. In fact, molluscs have been widely recommended for biomonitoring PTMs in coastal areas [84,85,86]. According to Phillips and Rainbow [84] and Rainbow [85], the metals accumulated in the tissues of molluscs are indicators of metal bioavailability in coastal waters.

An edible mollusc that is ideal for biomonitoring sea shelf pollution is the snail *Rapana venosa* [42]. Yap et al. [64] used linear regression to determine that there are positive and significant connections between the soft tissues of *P. viridis* and their habitat sediments for Cd, Cu, and Pb. As a result, it has been determined that the soft tissues of *P. viridis* are a possible biomonitor for Cd, Cu and Pb.

Yap et al. [87] demonstrated the possible use of *C. obtusa* soft tissues as a suitable biomonitor of Mn and Sc, based on the positive connection between the soft tissue and the habitat sediments. They did this by utilizing a straightforward linear regression equation. The bioaccumulation of PTMs by gastropods is controlled by environmental parameters such as salinity, pH, food availability and sediment quality [88], even though gastropods are a biomonitor.

Therefore, for optimal mangrove management in light of the sustainable resources from the intertidal mangrove environment, *C. obtusa* can be suggested as a potential biomonitor of PTM contamination in the mangrove ecosystem.

### 4.3. Health Risk–Biomonitoring Nexus versus Seafood–Water–Energy Nexus

**Figure 6:**
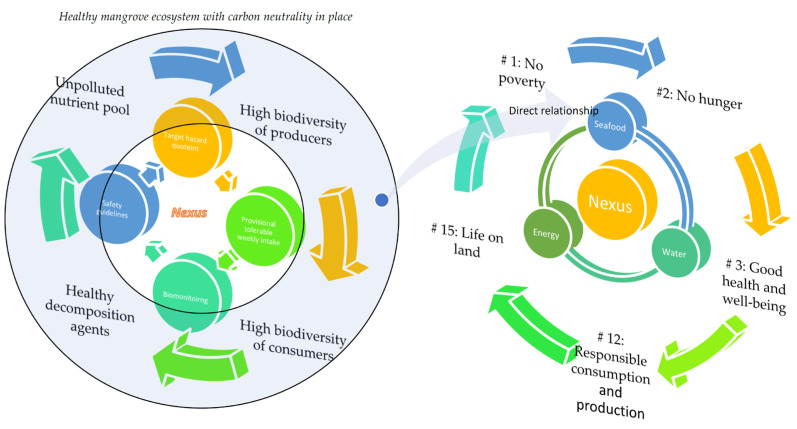
Conceptual relationship between health risks–biomonitoring nexus versus the seafood–water–energy nexus.

The relationship between the biomonitoring–health risk nexus versus the seafood–water–energy nexus is presented in Figure 6. The cycle on the left side of Figure 6 begins with the ideal ecosystem nutrient cycling, consisting of a healthy cycle of a nutrient–autotroph–heterotroph–decomposer nexus under the optimum energy empowered by the natural and without-haze atmospheric sunlight. This would help to support the supply chain of the biomonitoring–safety guidelines–THQ–PTWI nexus. The natural capital will bring direct support to the seafood–energy–water nexus in which a good quality and quantity of seafood supply can be expected. This is in tandem with the expected expansion of the worldwide human population. Of the 17 United Nations’ Sustainable Development Goals, Goal 1 (no poverty), Goal 2 (no hunger), Goal 3 (good health and well-being), Goal 12 (responsible consumption and production) and Goal 15 (life on land), are considered integrated into and will benefit from the above-described conceptual relationship nexus.

The exponential energy and water demand increase associated with this worldwide transformation has increased concerns over resource security [89]. Continuous evaluation of the potential dangers of PTMs to human health in the mangrove ecosystem is necessary for mitigation strategies to lessen the severity of the depletion and its environmental implications. This study concentrated on the safety of snails, which might be a key factor in the supply chain for sustainable and high-quality seafood products. It also considered biomonitoring and health risk assessments of the PTMs of the snail resources along the chain (Figure 6). This knowledge can help the supply chain of the snail seafood industry attain sustainability via cogent policymaking and management [89].

The concept of the seafood–energy–water nexus in relation to seafood consumption has been discussed in the literature, such as studies from China [89,90] and Scotland [91]. For instance, Qu et al. [91] suggested that expanding the number of offshore wind farms (OWFs) will have a detrimental, although limited, impact on industries that produce seafood through economic linkages. However, the declining energy cost from OWFs would assist lower-income people and benefit the social economy as a whole, helping to reduce fuel poverty. Their findings, therefore, underline the need to understand the connections between OWFs and seafood production.

The present proposed biomonitoring–safety guidelines–THQ–PTWI nexus is consistent with the Food Safety Objective approach, which is the core principle of process validation [92]. All the aforementioned biomonitoring data indicate that the product quality of snail seafood should be regularly monitored for PTM contamination with particular reference to human health risk assessments of PTMs in the snails collected from the resourceful yet human-impacted mangrove ecosystem.

It is not exaggerated to also point out that the proposed biomonitoring–safety guidelines–THQ–PTWI nexus can be a trigger and a driving force for the momentum of fast development over the long term which focuses on the mangrove ecosystem. This is because the seafood—energy–water nexus is an essential economic input and also an indispensable criterion for global economic development [93]. Surely, seafood is a crucial natural resource from the coastal mangrove for human survival and economic growth. Comprehensive information on seafood safety and the sustainability of the water–seafood–energy nexus at a global scale in terms of heavy metal contamination is urgently required [93].

To increase the social and environmental sustainability of electric and electronic equipment manufacturing in Africa, which has grown to be the primary source of PTM pollution, Garcia-Vazquez et al. [94] emphasized the research priorities that should be addressed. The major water-use processes that should be taken into account for seafood production were also emphasized by Gephart et al. [95] by reviewing water-use principles through a focus on seafood production systems. They provided a new perspective on the study of water use in food systems as a whole. This is because the environmental effects of both careless overfishing and habitat loss now contribute to a shortage of seafood and future food insecurity [96].

In summary, protecting the mangrove ecosystem is important for controlling pollution from PTMs that inhabit the local seafood gastropods. This can be achieved by proposing the biomonitoring of health risks of PTMs in the mangrove snails.

## 5. Conclusions

The present results clearly show that there is no health concern associated with the consumption of Malaysian snails, although the health risks depend on the consumers’ body weight and consumption rate. In sum, the current findings suggest that the levels of snail consumption should be restricted to reduce any potential health hazards for consumers associated with PTMs. *C. obtusa* may be a possible biomonitor for Cu, Ni, Pb and Zn, based on the relatively low and weak but positive correlations of Cu, Ni, Pb and Zn between this species and the sediments of its habitat. This is crucial for good mangrove management in light of the intertidal environment’s sustainable resources. Therefore, from the current investigation, the biomonitoring–health risk nexus of PTMs on mangrove snails is suggested.

## Figures and Tables

**Table 1:** Overall statistics of concentrations (mg/kg dry weight (dw)) and those converted into a wet weight (ww) basis of six potential toxic metals in the total soft tissues of 21 populations of *C. obtusa* collected from the west mangrove coast of Peninsular Malaysia. N = 21.

	Cd_dw_	Cd_ww_	Cu_dw_	Cu_ww_	Fe_dw_	Fe_ww_
Min	0.11	0.03	47.6	11.4	171	40.9
Max	9.65	2.32	147	35.2	3162	759
Mean	2.05	0.49	103	24.7	600	144
Median	1.22	0.29	103	24.8	356	85.4
SD	2.42	0.58	25.6	6.15	681	163
SE	0.53	0.13	5.59	1.34	149	35.7
Skewness	1.92	1.93	−0.58	−0.58	2.83	2.83
Kurtosis	3.20	3.22	−0.13	−0.13	7.81	7.81
	Ni_dw_	Ni_ww_	Pb_dw_	Pb_ww_	Zn_dw_	Zn_ww_
Min	1.67	0.4	3.75	0.90	12.9	3.11
Max	25.6	6.14	55.9	13.4	536	129
Mean	10.1	2.43	15.1	3.63	146	35.1
Median	8.85	2.12	10.8	2.6	105	25.2
SD	6.57	1.58	12.3	2.96	121	28.9
SE	1.43	0.34	2.69	0.65	26.3	6.31
Skewness	0.90	0.90	1.89	1.90	2.33	2.33
Kurtosis	−0.04	−0.04	3.77	3.78	4.59	4.59

Note: SE = standard error; SD = standard deviation.

**Table 2:** Overall statistics of values of estimated daily intake (EDI, µg/kg body weight/day) and target hazard quotient (THQ) for six potential toxic metals in the total soft tissues of 21 populations of *Cerithidea obtusa* collected in the present study. N = 21.

	Cd EDI	Cd THQ	Cu EDI	Cu THQ	Ni EDI	Ni THQ
Min	0.02	0.02	7.37	0.18	0.26	0.013
Max	1.49	1.49	22.7	0.57	3.96	0.198
Mean	0.32	0.32	15.9	0.40	1.56	0.08
Median	0.19	0.19	15.9	0.4	1.37	0.069
SD	0.38	0.38	3.97	0.10	1.02	0.05
SE	0.08	0.08	0.87	0.02	0.22	0.01
Skewness	1.92	1.92	−0.58	−0.58	0.90	0.90
Kurtosis	3.20	3.20	−0.13	−0.13	−0.04	−0.04
	Fe EDI	Fe THQ	Pb EDI	Pb THQ	Zn EDI	Zn THQ
Min	26.4	0.04	0.58	0.16	2.01	0.01
Max	490	0.70	8.65	2.47	83.0	0.28
Mean	92.9	0.13	2.34	0.67	22.7	0.08
Median	55.1	0.08	1.68	0.48	16.3	0.05
SD	105	0.15	1.91	0.54	18.7	0.06
SE	23.0	0.03	0.42	0.12	4.07	0.01
Skewness	2.83	2.83	1.89	1.89	2.33	2.34
Kurtosis	7.81	7.80	3.77	3.77	4.59	4.61

Note: SE = standard error; SD = standard deviation.

**Table 3:** Overall statistics of values of estimated weekly intake (EWI, µg/kg body weight/week) and their percentages in comparison to provisional tolerable weekly intake (PTWI, µg/kg body weight/week) of six potential toxic metals in the total soft tissues of 21 populations of *Cerithidea obtusa* collected in the present study. N = 21.

N = 21	Cd EWI	Cu EWI	Ni EWI	Fe EWI	Pb EWI	Zn EWI
Min	0.12	51.5	1.81	185	4.06	14.1
Max	10.46	159	27.7	3427	60.6	581
Mean	2.22	112	10.9	651	16.4	159
Median	1.32	112	9.59	386	11.8	114
SD	2.63	27.8	7.12	738	13.4	131
SE	0.57	6.06	1.55	161	2.91	28.5
Skewness	1.93	−0.58	0.90	2.83	1.90	2.33
Kurtosis	3.21	−0.13	−0.04	7.81	3.78	4.59
	Cd%EWI	Cu%EWI	Ni%EWI	Fe%EWI	Pb%EWI	Zn%EWI
Min	0.033	0.024	0.032	0.053	0.31	0.003
Max	2.893	0.073	0.492	0.987	4.65	0.134
Mean	0.61	0.05	0.19	0.19	1.26	0.04
Median	0.364	0.052	0.17	0.111	0.90	0.026
SD	0.73	0.01	0.13	0.21	1.03	0.03
SE	0.16	0.00	0.03	0.05	0.22	0.01
Skewness	1.92	−0.61	0.90	2.83	1.90	2.33
Kurtosis	3.20	−0.12	−0.04	7.80	3.77	4.59

Note: SE = standard error; SD = standard deviation.

## Data Availability

Not applicable.

## Supplementary Materials

The following supporting information can be downloaded at: https://www.mdpi.com/article/10.3390/foods12081575/s1; Table S1: Sampling dates, locations and shell lengths (SL) of *Cerithedea obtusa* collected from the present study; Table S2: A comparison of trace metal concentrations (mean ± SE, µg/kg dry weight) between measured values and certified values in the Certified Reference Materials (CRM) for mussel tissue (NIST 2976), Dogfish Liver (DOLT-3, National Research Council Canada); Table S3: Values of oral reference dose (ORD, µg/kg body weight/day), and provisional tolerable weekly intakes (PTWI, mg/kg body weight/week) in the six potentially toxic metals used in the present study; Table S4: Mean concentrations (mg/kg dry weight dry weight) and those converted into wet basis of 6 potential toxic metals in the total soft tissues of *Cerithidea obtusa* populations collected from the present study; Table S5: Values of estimated daily intake (EDI, µg/kg body weight/day), target hazard quotient (THQ), estimated weekly intake (EWI, µg/kg body weight/day) for of 6 potential toxic metals in the total soft tissues of 21 populations of *Cerithidea obtusa* collected from the present study; Table S6: Values of estimated weekly intake (EWI, µg/kg body weight/day) and their percentages in comparison to provisional tolerable weekly intake (PTWI) for of 6 potential toxic metals in the total soft tissues of 21 populations of *Cerithidea obtusa* collected from the present study.

## References

[1] Haseeb-ur-Rehman M., Munshi A.B., Atique U., Kalsoom S. (2023). Metal pollution and potential human health risk assessment in major seafood items (fish, crustaceans, and cephalopods). Mar. Pollut. Bull..

[2] Tanhan P., Lansubsakul N., Phaochoosak N., Sirinupong P., Yeesin P., Imsilp K. (2023). Human Health Risk Assessment of Heavy Metal Concentration in Seafood Collected from Pattani Bay, Thailand. Toxics.

[3] Rodrigues P.D.A., Ferrari R.G., do Rosário D.K.A., de Almeida C.C., Saint’Pierre T.D., Hauser-Davis R.A., dos Santos L.N., Conte-Junior C.A. (2022). Toxic metal and metalloid contamination in seafood from an eutrophic Brazilian estuary and associated public health risks. Mar. Pollut. Bull..

[4] Petrovic J., Jovetic M., Štulić M., Vujadinović D., Lorenzo J.M., Iammarino M., Djekic I.V., Tomasevic I. (2022). Exposure assessment in the Serbian population and occurrence of histamine and heavy metals in fish and seafood. Int. J. Food Sci. Technol..

[5] Qi Z., Cao H., Hu Y., Du M., Pan Y., Zhao Y., Liu H. (2022). Differences and risk assessment of heavy metals in seafood and freshwater products. J. Fish China.

[6] Patrick-Iwuanyanwu K.C., Esau B.S., Ogbo B.A., Egbuna C., Ibeabuchi C.G., Orajiaka-Uchegbu C. (2022). Health Risk Assessment of Hazardous Metals in Seafood from Ka-Bangha River, Khana, Rivers State, Nigeria. Egypt. J. Aquat. Biol. Fish..

[7] Pandion K., Khalith S.B.M., Ravindran B., Chandrasekaran M., Rajagopal R., Alfarhan A., Chang S.W., Ayyamperumal R., Mukherjee A., Arunachalam K.D. (2022). Potential health risk caused by heavy metal associated with seafood consumption around coastal area. Environ. Pollut..

[8] Bu-Olayan A.H., Thomas B.V. (2001). Heavy metal accumulation in the gastropod, *Cerithium scabridum* l.; from the Kuwait Coast. Environ. Monit. Assess..

[9] Salam M.A., Dayal S.R., Siddiqua S.A., Muhib M.I., Bhowmik S., Kabir M.M., Rak A.A.L.E., Srzednicki G. (2021). Risk assessment of heavy metals in marine fish and seafood from Kedah and Selangor coastal regions of Malaysia: A high-risk health concern for consumers. Environ. Sci. Pollut. Res..

[10] Boulajfene W., Strogyloudi E., Catsiki V.-A., El Mlayah A., Tlig-Zouari S. (2017). Bio-monitoring of metal impact on metallothioneins levels in the gastropod *Phorcus turbinatus* (Born, 1778) in the northeastern and the eastern coasts of Tunisia. Mar. Pollut. Bull..

[11] Da Le N., Ha Hoang T.T., Phung V.P., Nguyen T.L., Duong T.T., Dinh L.M., Huong Pham T.M., Binh Phung T.X., Nguyen T.D., Duong T.N. (2021). Trace Metal Element Analysis in Some Seafood in the Coastal Zone of the Red River (Ba Lat Estuary, Vietnam) by Green Sample Preparation and Inductively Coupled Plasma-Mass Spectrometry (ICP-MS). J. Anal. Meth. Chem..

[12] Djedjibegovic J., Marjanovic A., Tahirovic D., Caklovica K., Turalic A., Lugusic A., Omeragic E., Sober M., Caklovica F. (2020). Heavy metals in commercial fish and seafood products and risk assessment in adult population in Bosnia and Herzegovina. Sci. Rep..

[13] Orajiaka-Uchegbu C., Patrick-Iwuanyanwu K.C., Ogbo A.B., Egbuna C. (2020). Bioaccumulation of heavy metals and potential health risk through consumption of seafoods from selected creeks in rivers state, Nigeria. Egypt. J. Aquat. Biol. Fish..

[14] Di Bella C., Traina A., Giosuè C., Carpintieri D., Lo Dico G.M., Bellante A., Del Core M., Falco F., Gherardi S., Uccello M.M. (2020). Heavy Metals and PAHs in Meat, Milk, and Seafood from Augusta Area (Southern Italy): Contamination Levels, Dietary Intake, and Human Exposure Assessment. Front. Publ. Health.

[15] Soceanu A., Dobrinas S., Carazeanu Popovici I., Jitariu D. (2020). Health risk assessment of heavy metals in seafood. J. Environ. Prot. Ecol..

[16] Milenkovic B., Stajic J.M., Stojic N., Pucarevic M., Strbac S. (2019). Evaluation of heavy metals and radionuclides in fish and seafood products. Chemosphere.

[17] Saher N.U., Kanwal N. (2019). Assessment of some heavy metal accumulation and nutritional quality of shellfish with reference to human health and cancer risk assessment: A seafood safety approach. Environ. Sci. Pollut. Res..

[18] Mazrouh M.M., Mourad M.H. (2019). Biochemical composition and bioaccumulation of heavy metals in some seafood in the mediterranean coast of Egypt. Egypt. J. Aquat. Biol. Fish..

[19] Zhao B., Wang X., Jin H., Feng H., Shen G., Cao Y., Yu C., Lu Z., Zhang Q. (2018). Spatiotemporal variation and potential risks of seven heavy metals in seawater, sediment, and seafood in Xiangshan Bay, China (2011–2016). Chemosphere.

[20] Lehel J., Bartha A., Dankó D., Lányi K., Laczay P. (2018). Heavy metals in seafood purchased from a fishery market in Hungary. Food Addit. Contam. Part B Surveil..

[21] Liu Q., Liao Y., Shou L. (2018). Concentration and potential health risk of heavy metals in seafoods collected from Sanmen Bay and its adjacent areas, China. Mar. Pollut. Bull..

[22] Kuplulu O., Iplikcioglu Cil G., Korkmaz S.D., Aykut O., Ozansoy G. (2018). Determination of metal contamination in seafood from the Black, Marmara, Aegean and Mediterranean sea metal contamination in seafood. J. Hell. Vet. Med. Soc..

[23] Satapathy S., Panda C.R. (2017). Toxic metal ion in seafood: Meta-analysis of human carcinogenic and non-carcinogenic threat assessment, a geomedical study from Dhamra and Puri, Odisha. Hum. Ecol. Risk Assess..

[24] Zhao R., Yan S., Liu M., Wang B., Hu D., Guo D., Wang J., Xu W., Fan C. (2016). Seafood consumption among Chinese coastal residents and health risk assessment of heavy metals in seafood. Environ. Sci. Pollut. Res..

[25] Nkpaa K.W., Patrick-Iwuanyanwu K.C., Wegwu M.O., Essien E.B. (2016). Health risk assessment of hazardous metals for population via consumption of seafood from Ogoniland, Rivers State, Nigeria; A case study of Kaa, B-Dere, and Bodo City. Environ. Monit. Assess..

[26] Yap C.K., Al-Mutairi K.A. (2022). Comparative study of potentially toxic nickel and their potential human health risks in seafood (fish and mollusks) from Peninsular Malaysia. Biology.

[27] Yap C.K. (2012). Mussel Watch in Malaysia Past, Present and Future.

[28] Joseph A., Iwok E., Ekanem S. (2021). Public health threats of heavy metals due to the consumption of *Achachatina marginata* (African giant land snail) from a partially remediated site in Ikot Ada Udo, Akwa Ibom State, South-South Nigeria. Environ. Pollut..

[29] Soegianto A., Putranto T.W.C., Payus C.M., Zarqasi F.R., Syafitrirulla P.P., Muchlisin M.I., Ramdhani S., Nosafandra A.S., Wibisono A.D. (2021). Metals in the tissues of the East Java Coast Indonesian green mussel (*Perna viridis* Linnaeus, 1758) and associated health risks. Reg. Stud. Mar. Sci..

[30] Montojo U.M., Baldoza B.J.S., Cambia F.D., Benitez K.C.D., Perelonia K.B.S., Rivera A.T.F. (2021). Levels and health risk assessment of mercury, cadmium, and lead in green mussel (*Perna viridis*) and oyster (*Crassostrea iredalei*) harvested around Manila Bay, Philippines. Food Control.

[31] Genchi G., Carocci A., Lauria G., Sinicropi M.S., Catalano A. (2020). Nickel: Human health and environmental toxicology. Int. J. Environ. Res. Public Health.

[32] Das K.K., Reddy R.C., Bagoji I.B., Das S., Bagali S., Mullur L., Khodnapur J.P., Biradar M.S. (2018). Primary concept of nickel toxicity—An overview. J. Basic Clin. Physiol. Pharmacol..

[33] Buxton S., Garman E., Heim K.E., Lyons-Darden T., Schlekat C.E., Taylor M.D., Oller A.R. (2019). Concise review of nickel human health toxicology and rcotoxicology. Inorganics.

[34] Garcia-Leston J., Mendez J., Pasaro E., Laffon B. (2010). Genotoxic effects of lead: An updated review. Environ. Int..

[35] Jovic M., Stankovic S. (2014). Human exposure to trace metals and possible public health risks via consumption of mussels *Mytilus galloprovincialis* from the Adriatic coastal area. Food Chem. Toxicol..

[36] Canfield R.L., Henderson C.R., Cory-Slechta D.A., Cox C., Jusko T.A., Lanphear B.P. (2003). Intellectual impairment in children with blood lead concentrations below 10 lg per deciliter. N. Engl. J. Med..

[37] Zhu F., Fan W., Wang X., Qu L., Yao S. (2011). Health risk assessment of eight heavy metals in nine varieties of edible vegetable oils consumed in China. Food Chem Toxicol..

[38] Gorell J.M., Johnson C.C., Rybicki B.A., Peterson E.L., Kortsha G.X., Brown G.G. (1997). Occupational exposures to metals as risk factors for Parkinson’s disease. Neurology.

[39] JECFA (1983). Evaluation of Certain Food Additives and Contaminants (Twenty-Seventh Report of the Joint FAO/WHO Expert Committee on Food Additives). https://apps.who.int/iris/handle/10665/39165.

[40] JECFA (1982). Evaluation of Certain Food Additives and Contaminants (Twenty-Sixth Report of the Joint FAO/WHO Expert Committee on Food Additives). https://apps.who.int/iris/handle/10665/41546.

[41] Mertz W. (1981). The essential trace elements. Science.

[42] Oyaro N., Juddy O., Murago E.N.M., Gitonga E. (2007). The contents of Pb, Cu, Zn and Cd in meat in Nairobi, Kenya. J. Food Agric. Environ..

[43] AFS (2021). Annual Fisheries Statistics of Malaysia Department of Fisheries Malaysia. https://www.dof.gov.my/en/resources/fisheries-statistics-i/.

[44] Amin B., Nurrachmi I. (2015). Concentration, distribution, and correlation of heavy metals in seawater, sediment, and *Cerithidea obtusa* from coastal waters of Singkep Island, Riau Archipelago Province. Indon. J. Environ. Sci. Technol..

[45] Duysak Ö., Azdural K. (2017). Evaluation of heavy metal and aluminium accumulation in a gastropod, *Patella caerulea* L.; 1758 in Iskenderun Bay, Turkey. Pak. J. Zool..

[46] Hamli H., Idris M.H., Hena M.A., Wong S.K. (2012). Diversity of edible mollusc (Gastropoda and Bivalvia) at selected divison of Sarawak, Malaysia. Int. J. Adv. Sci. Eng. Inform. Technol..

[47] Idris M.H., Hamli H., Kamal A.H.M., Lah R.A., Jaafar N.M.S.N. (2021). Study of diversity and morphometry in edible bivalves and gastropods from a coastal wetland in Sarawak. Songklanakarin J. Sci. Technol..

[48] Joseph T.U.R., Ramesh K.B. (2016). Heavy Metal Risk Assessment in Bhavanapadu Creek Using Three Potamidid Snails-*Telescopium telescopium*, *Cerithidea obtusa* and *Cerithidea cingulata*. J. Environ. Anal. Toxicol..

[49] Krishnan K., Saion E., Yap C.K., As N. (2022). Bioaccumulation of Metals in Mangrove Snail (*Cerithidea obtusa*) from Southwest Johor, Malaysia. Environ. Ecol. Res..

[50] Kumar K., Saion E., Yap C.K., Balu P., Cheng W.H., Chong M.Y. (2022). Distribution of heavy metals in sediments and soft tissues of the *Cerithidea obtusa* from Sepang River, Malaysia. Indon. J. Chem..

[51] Liu X., Yu S., Chen P., Hong B., Zhang Y., Lin X., Ma T., Zhou T., Li Y. (2022). Metal loadings in estuarine bivalve and gastropod shellfish in response to socio-economic development in watershed. Mar. Environ. Res..

[52] Menon M., Mohanraj R.V.B.J., Prasath R.V.A. (2023). Bioaccumulation of heavy metals in a gastropod species at the Kole wetland agroecosystem, a Ramsar site. J. Environ. Manag..

[53] Primost M.A., Gil M.N., Bigatti G. (2017). High bioaccumulation of cadmium and other metals in Patagonian edible gastropods. Mar. Biol. Res..

[54] Ragi A.S., Leena P.P., Cheriyan E., Nair S.M. (2017). Heavy metal concentrations in some gastropods and bivalves collected from the fishing zone of South India. Mar. Pollut. Bull..

[55] Ryabushko V.I., Toichkin A.M., Kapranov S.V. (2022). Heavy Metals and Arsenic in Soft Tissues of the Gastropod Rapana venosa (Valenciennes, 1846) Collected on a Mollusk Farm Off Sevastopol (Southwestern Crimea, Black Sea): Assessing Human Health Risk and Locating Regional Contamination Areas. Bull. Environ. Contam. Toxicol..

[56] Sumanti S., Siregar Y.I. (2019). Annalysis of Pb, Cu and Zn Metal Contents in Red Chut-chut Snail (*Cerithidea obtusa*) and Sediment in Mendol Island Kuala Kampar of Riau Province. Asian J. Aquat. Sci..

[57] Thanh-Nho N., Marchand C., Strady E., Huu-Phat N., Nhu-Trang T.T. (2019). Bioaccumulation of some trace elements in tropical mangrove plants and snails (Can Gio, Vietnam). Environ. Pollut..

[58] Wariski I., Siregar Y.I., Amin B. (2021). Heavy Metal Content in Sediment and Hora Shell (*Cerithidea obtusa*) in Panipahan Waters, Rokan Hilir, Riau. J. Nat. Indon..

[59] Yap C.K., Edward F.B. (2010). Distribution of heavy metals in the different parts of *Cerithidea obtusa* and the relationships between metal distribution and allometric parameters of the snail. Environ. Asia.

[60] Yap C.K., Edward F.B. (2009). Heavy metal distribution in the different parts of *Cerithidea obtusa* by using multivariate analysis. Malay. J. Sci..

[61] Cheng W.H., Yap C.K. (2015). Potential human health risks from toxic metals via mangrove snail consumption and their ecological risk assessments in the habitat sediment from Peninsular Malaysia. Chemosphere.

[62] Artalina D., Takarina N.D. (2019). Metals Content in Edible Gastropod from Blanakan Silvofishery Ponds. J. Phys. Conf. Ser..

[63] Yap C.K., Hisyam M.N.D., Edward F.B., and Tan S.G. (2010). Concentrations of heavy metal in the different parts of gastropod, *Faunus ater* collected from the intertidal area of Peninsular Malaysia. Pertanika J. Trop. Agric. Sci..

[64] Yap C.K., Ismail A., Tan S.G., Omar H. (2002). Correlations between speciation of Cd, Cu, Pb and Zn in sediment and their concentrations in total soft tissue of green-lipped mussel *Perna viridis* from the west coast of Peninsular Malaysia. Environ. Int..

[65] US FDA/CFSAN National Shellfish Sanitation Program Guide for the Control of Molluscan Shellfish. Guidance Documents Chapter II. Growing Areas: 04. Action Levels, Tolerances, and Guidance Levels for Poisonous or Deleterious Substances in Seafood, 2007 (2019 Revised Edition). https://www.fda.gov/media/143238/download.

[66] Food Safety and Quality Division of the Ministry of Health Malaysia (1985). Malaysian Law on Food and Drugs.

[67] European Commission (2006). Commission Regulation (EC) No 1881/2006 of the European Parliament and the Council of 19 December 2006 Setting Maximum Levels for Certain Contaminants In Foodstuffs. Official Journal of the European Communities, L364/18. http://eur-lex.europa.eu/legal-content/EN/TXT/PDF/?uri=CELEX:32006R1881&from=EN.

[68] Codex Alimentarius (1997). International Food Standard. General Standard for Contaminants and Toxins in Food and Feed. Cxs 193-1995. Adopted in 1995 Revised in 1997, 2006, 2008 and 2009, Amended in 2010, 2012–2019. FAO and WHO. 66 pages. https://www.fao.org/fao-who-codexalimentarius/sh-proxy/en/?lnk=1&url=https%253A%252F%252Fworkspace.fao.org%252Fsites%252Fcodex%252FStandards%252FCXS%2B193-1995%252FCXS_193e.pdf.

[69] ANZFA (2015). Australian and New Zealand Food Standards Code, Standard 1.4.1—Contaminants and Natural Toxicants (F2011C00052). https://www.comlaw.gov.au/Details/F2015C00052/Download.

[70] Nauen C. (1983). Compilation of Legal Limits for Hazardous Substances in Fish and Fishery Products. FAO Fisheries Circular, No. 464. https://agris.fao.org/agris-search/search.do?recordID=XF19840038090.

[71] Ahmad N.I., Wan Mahiyuddin W.R., Tengku Mohamad T.R., Ling C.Y., Daud S.F., Hussein N.C., Abdullah N.A., Shaharudin R., Sulaiman L.H. (2016). Fish Consumption Pattern among Adults of Different Ethnics in Peninsular Malaysia. Food Nutr. Res..

[72] US EPA Human Health Risk Assessment.; Regional Screening Level (RSL)—Summary Table November 2021. https://semspub.epa.gov/work/HQ/401635.pdf.

[73] JECFA (2010). Summary and Conclusions of the Seventy-Third Meeting of the JECFA..

[74] WHO (1993). Guidelines for Drinking-Water Quality: Volume 1, Recommendations.

[75] JECFA (2021). Evaluations of the Joint FAO/WHO Expert Committee on Food Additives (JECFA). Includes All Updates Up to the 89th JECFA (June 2020). https://apps.who.int/food-additives-contaminants-jecfa-database/search.aspx?fcc=2.

[76] Schrenk D., Bignami M., Bodin L., Chipman J.K., del Mazo J., Grasl-Kraupp B., Hogstrand C., Hoogenboom L., Leblanc J.C., EFSA Panel on Contaminants in the Food Chain (CONTAM) (2020). Update of the Risk Assessment of Nickel in Food and Drinking Water. EFSA J..

[77] JECFA (2011). Safety Evaluation of Certain Contaminants in Food/Prepared by the Seventy-Second Meeting of the Joint FAO/WHO Expert Committee on Food Additives (JECFA). https://apps.who.int/iris/handle/10665/44520.

[78] Zar J.H. (1996). Biostatistical Analysis, 3rd ed.

[79] Horiguchi H., Oguma E., Sasaki S., Miyamoto K., Ikeda Y., Machida M., Kayama F. (2004). Dietary exposure to cadmium at close to the current provisional tolerable weekly intake does not affect renal function among female Japanese farmers. Environ. Res..

[80] Oberdorster G.I. (1992). Pulmonary deposition, clearance and effects of inhalation soluble and insoluble cadmium compounds. IARC Sci. Publ..

[81] Horiguchi H., Oguma E., Sasaki S., Okubo H., Murakami K., Miyamoto K., Hosoi Y., Murata K., Kayama F. (2013). Age-relevant renal effects of cadmium exposure through consumption of home-harvested rice in female Japanese farmers. Environ. Int..

[82] Horiguchi H., Aoshima K., Oguma E., Sasaki S., Miyamoto K., Hosoi Y., Katoh T., Kayama F. (2010). Latest status of cadmium accumulation and its effects on kidneys, bone, and erythropoiesis in inhabitants of the formerly cadmium-polluted Jinzu River Basin in Toyama, Japan, after restoration of rice paddies. Int. Arch. Occup. Environ. Health.

[83] Institute of Medicine (2001). Dietary Reference Intakes for Vitamin A, Vitamin K, Arsenic, Boron, Chromium, Copper, Iodine, Iron, Manganese, Molybdenum, Nickel, Silicon, Vanadium, and Zinc.

[84] Phillips D.J.H., Rainbow P.S. (1989). Strategies of trace metals sequestration in aquatic organisms. Mar. Environ. Res..

[85] Rainbow P.S. (1997). Trace metal accumulation in marine invertebrates: Marine biology or marine chemistry. J. Mar. Biol. Assoc. UK.

[86] Phillips D.J.H. (1995). The chemistries and environmental fates of trace metals and organchlorines in aquatic ecosystems. Mar. Pollut. Bull..

[87] Yap C.K., Kumar K., Saion E., Kamari H.M., Wong K.W., Saito M., Nulit R., Shohaimi S., Bakhtiari A.R., Al-Shami S.A. (2018). Association of Trace Metals (Cr, Co, Mn and Sc) between Surface Sediments and the Mangrove Snail Cerithedea Obtusa: Assessment of the Snail as a Biomonitor for Intertidal Mangrove Ecosystem Management.

[88] Srivastava A.K., Singh V.K. (2020). Snails as biological monitor (bioindicator). Asian J. Adv. Res..

[89] Liu G., Arthur M., Viglia S., Xue J., Meng F., Lombardi G.V. (2020). Seafood-energy-water nexus: A study on resource use efficiency and the environmental impact of seafood consumption in China. J. Clean. Prod..

[90] Lu J., Lin Y., Wu J., Zhang C. (2021). Continental-scale spatial distribution, sources, and health risks of heavy metals in seafood: Challenge for the water-food-energy nexus sustainability in coastal regions?. Environ. Sci. Pollut. Res..

[91] Qu Y., Hooper T., Swales J.K., Papathanasopoulou E., Austen M.C., Yan X. (2021). Energy-food nexus in the marine environment: A macroeconomic analysis on offshore wind energy and seafood production in Scotland. Energy Policy.

[92] Keener L. (2022). Food Safety Objectives: The Nexus among Preventive Controls, Validation, and Food Safety Assurance. Food Safety Magazine. https://www.food-safety.com/articles/8336-food-safety-objectives-the-nexus-among-preventive-controls-validation-and-food-safety-assurance.

[93] Orimoloye I.R. (2022). Water, energy and food nexus: Policy relevance and challenges. Front. Sustain. Food Syst..

[94] Garcia-Vazquez E., Geslin V., Turrero P., Rodriguez N., Machado-Schiaffino G., Ardura A. (2021). Oceanic karma? Eco-ethical gaps in African EEE metal cycle may hit back through seafood contamination. Sci. Total Environ..

[95] Gephart J.A., Troell M., Henriksson P.J.G., Beveridge M.C.M., Verdegem M., Metian M., Mateos L.D., Deutsch L. (2017). The ‘seafood gap’ in the food-water nexus literature—Issues surrounding freshwater use in seafood production chains. Adv. Wat. Res..

[96] Nylen N.G. (2013). Why federal dietary guidelines should acknowledge the food-choice/Environment Nexus: Examining the recommendation to eat more seafood. Ecol. Law Q..

