# Supplementary material for: Biomonitoring–Health Risk Nexus of Potentially Toxic Metals on Cerithidea obtusa: A Biomonitoring Study from Peninsular Malaysia
Source: Foods. 2023 Apr 7;12(8):1575. doi: 10.3390/foods12081575 (PMC10138110; doi:10.3390/foods12081575)
Supplement: Supplementary file 1 [file foods-12-01575-s001.zip › foods-2312634-supplementary.pdf]

Supplementary Materials

Article

Biomonitoring–Health Risk Nexus of Potentially Toxic Metals on *Cerithidea obtusa*: A Biomonitoring Study from Peninsular Malaysia

Chee Kong Yap [1,*] and Khalid Awadh Al-Mutairi [2]

[1] Department of Biology, Faculty of Science, Universiti Putra Malaysia (UPM), Serdang 43400, Malaysia
[2] Department of Biology, Faculty of Science, University of Tabuk, Tabuk P.O. Box 741, Saudi Arabia;
kmutairi@ut.edu.sa
* Correspondence: yapchee@upm.edu.my

Table S1: Sampling dates, locations and shell lengths (SL) of *Cerithedea obtusa* collected from the present study.

No.	Sites		Date	N	E	SL (mm)
1	Sepang Besar-1	SB-1	1-Dec-07	N 02°36.653'	E 101°42.345'	32.7
2	Sepang Besar-2	SB-2	1-Dec-07	N 02°38.933	E 101°42.826'	41.7
3	Klang-1	KL-1	2-Dec-07	N 03º01.343′	E 101º22.511′	45.4
4	Klang-2	KL-2	2-Dec-07	N 03º01.546′	E 101º22.680′	45.3
5	Klang-3	KL-3	2-Dec-07	N 03º01.044′	E 101º20.560′	49.3
6	Klang-4	KL-4	2-Dec-07	NA	NA	46.2
6	Klang-5	KL-5	2-Dec-07	N 03º01.615′	E 101º21.150′	44.6
8	Tanjung kupang	TK	19-Jun-10	N 1°22.694"	E 103°38.094"	NA
9	Kuala Sg. Ayam	KSA	18-Jun-10	N 2°34.423"	E 102°02.346"	NA
10	Sepang Besar-3	SB-3	5-May-10	N 2°36.230"	E 101°42.139"	NA
11	Lukut-1	LK-1	27-Dec-10	N 2°35.153"	E 101°49.548"	NA
12	Lukut-2	LK-2	27-Dec-10	N 2°35.958"	E 101°47.509"	NA
13	Deralik, Sitiawan	DR	25-Feb-06	NA	NA	NA
14	Bako, Sarawak	BK	28-Dec-06	NA	NA	NA
15	Sematan, Sarawak	SM	28-Dec-06	NA	NA	NA
16	Muar Estuary	MR	8-May-07	02°09.573′	102°19.033′	36.3
17	Lukut-3	LK-3	1-Dec-07	N 02°34.978' ,	E 101°47.607'	41.6
18	Lukut-4	LK-4	1-Dec-07	N 02°34.847' ,	E 101°47.795'	43.4
19	Lukut-5	LK-5	1-Dec-07	N 02°34.853' ,	E 101°47.882'	38.0
20	Belanak, Juru	BL	7-Dec-07	NA	NA	45.3
21	Teluk Ayer Tawar, Juru	TAT	7-Dec-07	NA	NA	43.3

Note: NA= not available.

Table S2: A comparison of trace metal concentrations (mean ± SE, µg/kg dry weight) between measured values and certified values in the Certified Reference Materials (CRM) for mussel tissue (NIST 2976), Dogfish Liver (DOLT-3, National Research Council Canada).

Metals	Certified values (C)	Measured value (M)	Percentage of recovery [(M/C) x 100]
	Mean	Mean	
Cu	31.2 ± 1.00	26.8 ± 0.25	85.93
Cd	19.4 ± 0.60	14.7 ± 0.34	75.67
Fe	1484 ± 57.0	1213 ± 10.7	81.77
Ni	2.72 ± 0.35	3.37 ± 0.20	123.90
Pb	37.4 ± 12.8	29.4 ± 0.33	78.56
Zn	86.6 ± 2.40	76.1 ± 0.81	99.90

Supplementary Materials

Table S3: Values of oral reference dose (ORD, μg/kg body weight/day), and provisional tolerable weekly intakes (PTWI, mg/kg body weight/week) in the six potentially toxic metals used in the present study.

Metal	ORD*	Intake	Reference	PTWI	PTWI for 62 kg adult
Zn	300	PTDI (mg/kg bw/day) of Zn was calculated from a PMTDI of 1.00 mg/kg BW/day	JECFA (1982, 2021)	1.00 mg/kg BW/day) x 7 days= 7.00 mg/kg BW/week	7.00 mg/kg BW/week x 62 kg= 434 mg/week (434000 μg/week).
Fe	700	A PMTDI of 0.80 mg/kg BW/day	JECFA (1983, 2021)	0.80 mg/kg BW/day) x 7 days= 5.60 mg/kg BW/week	5.60 mg/kg BW/week x 62 kg= 347.2 mg/week (347200 μg/week).
Cu	40.0	A PMTDI of 0.50 mg/kg BW/day	JECFA (1982, 2021)	0.50 mg/kg BW/day) x 7 days= 3.50 mg/kg BW/week.	3.50 mg/kg BW/week x 62 kg= 217 mg/week (217000 μg/week).
Ni	20.0	A TDI of 13 μg/kg BW	EFSA (2020)	13 μg/kg BW x 7 days= 91 μg/kg BW	91 μg/kg BW x 62 kg= 5642 μg/week
Pb	3.50	For adults, the TDI (μg/kg bw/day) of Pb was calculated from the higher end of the range (3 μg/kg BW/day).	JECFA (2011)	3.00 μg/kg BW/day) x 7 days= 21.0 μg/kg BW/week	21.0 μg/kg BW/week x 62 kg= 1302 μg/week.
Cd	1.00	A PTMI of 25.0 μg/kg BW based on a month of 30 day. Therefore, PTDI= (25 μg/kg BW/month)/ 30 days= 0.833 μg/kg BW/day	JECFA (2011, 2021)	0.833 μg/kg BW/day) x 7 days= 5.83 μg/kg BW/week	5.83 μg/kg BW/week x 62 kg= 361.5 μg/week

Note: *= The values of oral reference dose (ORD) for all metals are specified by the US EPA regional screening level (USEPA, 2021). BW= Body weight. TDI= Tolerable daily intake; PTMI= provisional tolerable monthly intake; PTDI= provisional tolerable daily intake. PMTDI= provisional maximum tolerable daily intake.

Table S4: Mean concentrations (mg/kg dry weight dry weight) and those converted into wet basis of 6 potential toxic metals in the total soft tissues of *Cerithidea obtusa* populations collected from the present study.

		Cd			Cu			Fe			Ni			Pb			Zn		
No.	Sites	Dry	Wet	SED	Dry	Wet	SED	Dry	Wet	SED	Dry	Wet	SED	Dry	Wet	SED	Dry	Wet	SED
1	SB-1	0.80	0.19	3.81	64.80	15.55	22.73	674	161.80	25512	9.35	2.24	13.12	13.77	3.30	25.55	105.01	25.20	82.13
2	SB-2	2.60	0.62	4.42	99.61	23.91	9.00	171	40.98	13564	4.43	1.06	8.49	4.12	0.99	29.13	88.53	21.25	64.89
3	KL-1	0.18	0.04	0.73	84.33	20.24	37.55	218	52.32	24175	10.74	2.58	15.75	14.23	3.41	45.86	96.63	23.19	158.90
4	KL-2	1.22	0.29	1.45	103.26	24.78	37.92	238	57.00	22121	3.87	0.93	15.94	10.32	2.48	48.57	100.24	24.06	168.27
5	KL-3	0.11	0.03	0.81	116.45	27.95	44.31	351	84.23	22533	4.12	0.99	18.46	19.79	4.75	54.09	132.27	31.74	182.94
6	KL-4	0.12	0.03	NA	126.30	30.31	NA	303	72.77	NA	6.61	1.59	NA	27.64	6.63	NA	94.16	22.60	NA
7	KL-5	0.45	0.11	0.72	136.02	32.64	53.99	344	82.46	23095	12.95	3.11	19.51	9.85	2.36	62.34	209.73	50.33	206.77
8	TK	4.41	1.06	0.47	114.67	27.52	14.70	216	51.75	47324	12.58	3.02	18.07	16.15	3.88	37.86	121.87	29.25	74.46
9	KSA	7.04	1.69	2.81	93.12	22.35	19.81	370	88.77	59281	12.94	3.11	24.66	33.80	8.11	164.95	125.22	30.05	115.99
10	SB-3	9.65	2.32	0.68	99.20	23.81	22.97	214	51.26	22157	20.77	4.98	4.26	55.86	13.41	31.99	158.27	37.98	26.47
11	LK-1	2.32	0.56	1.22	47.60	11.42	17.28	336	80.70	41039	16.31	3.91	7.87	6.06	1.45	25.16	92.93	22.30	34.78
12	LK-2	2.97	0.71	1.51	53.41	12.82	32.92	659	158.18	75804	25.59	6.14	17.66	3.75	0.90	40.43	110.33	26.48	89.14
13	DR	1.53	0.37	3.07	111.58	26.78	11.02	483	115.80	15315	11.25	2.70	5.99	19.78	4.75	28.84	108.53	26.05	33.46
14	BK	1.29	0.31	NA	124.20	29.81	NA	356	85.44	NA	7.53	1.81	NA	7.06	1.69	NA	84.73	20.33	NA
15	SM	1.15	0.28	NA	128.10	30.74	NA	207	49.68	NA	7.37	1.77	NA	5.35	1.28	NA	99.40	23.86	NA
18	MR	0.28	0.07	0.81	146.55	35.17	22.96	815	195.59	34839	8.85	2.12	15.28	4.91	1.18	53.90	181.12	43.47	118.65
17	LK-3	0.58	0.14	2.74	92.02	22.08	13.78	360	86.38	25728	1.67	0.40	10.11	7.29	1.75	28.93	96.89	23.25	45.52
18	LK-4	0.29	0.07	2.87	91.14	21.87	11.25	431	103.55	24556	2.95	0.71	11.53	7.90	1.90	41.68	81.98	19.68	74.29
19	LK-5	0.88	0.21	2.81	119.42	28.66	9.37	1112	266.81	21200	5.26	1.26	11.23	10.84	2.60	44.76	12.96	3.11	61.96
20	BL	2.88	0.69	1.1	113.23	27.18	24.6	3162	758.84	28783	21.98	5.28	20.24	19.79	4.75	32.05	536.25	128.70	140.39
21	TAT	2.24	0.54	0.98	97.51	23.40	18.54	1590	381.72	23810	5.09	1.22	21.4	19.57	4.70	36.73	435.72	104.57	107.27

Note: The metal data in dry weight basis were converted into wet weight ones by using 0.24. NA= data not available.

Table S5: Values of estimated daily intake (EDI, µg/kg body weight/day), target hazard quotient (THQ), estimated weekly intake (EWI, µg/kg body weight/day) for of 6 potential toxic metals in the total soft tissues of 21 populations of *Cerithidea obtusa* collected from the present study.

Sites	Cd TST EDI	Cd TST THQ	Cu TST EDI	Cu TST THQ	Ni TST EDI	Ni TST THQ	Fe TST EDI	Fe TST THQ	Pb TST EDI	Pb TST THQ	Zn TST EDI	Zn TST THQ
SB-1	0.124	0.124	10.033	0.251	1.447	0.072	104.386	0.149	2.132	0.609	16.260	0.054
SB-2	0.402	0.402	15.423	0.386	0.686	0.034	26.439	0.038	0.638	0.182	13.708	0.046
KL-1	0.028	0.028	13.058	0.326	1.663	0.083	33.755	0.048	2.203	0.629	14.961	0.050
KL-2	0.188	0.188	15.988	0.400	0.599	0.030	36.777	0.053	1.598	0.457	15.521	0.052
KL-3	0.017	0.017	18.030	0.451	0.637	0.032	54.343	0.078	3.064	0.876	20.480	0.068
KL-4	0.018	0.018	19.557	0.489	1.024	0.051	46.951	0.067	4.280	1.223	14.579	0.049
KL-5	0.069	0.069	21.060	0.527	2.004	0.100	53.197	0.076	1.526	0.436	32.474	0.108
TK	0.683	0.683	17.755	0.444	1.948	0.097	33.386	0.048	2.501	0.714	18.870	0.063
KSA	1.090	1.090	14.419	0.360	2.004	0.100	57.272	0.082	5.233	1.495	19.389	0.065
SB-3	1.494	1.494	15.360	0.384	3.216	0.161	33.072	0.047	8.649	2.471	24.506	0.082
LK-1	0.360	0.360	7.370	0.184	2.525	0.126	52.066	0.074	0.938	0.268	14.388	0.048
LK-2	0.460	0.460	8.270	0.207	3.963	0.198	102.055	0.146	0.580	0.166	17.083	0.057
DR	0.236	0.236	17.276	0.432	1.742	0.087	74.710	0.107	3.062	0.875	16.804	0.056
BK	0.199	0.199	19.231	0.481	1.165	0.058	55.123	0.079	1.092	0.312	13.119	0.044
SM	0.178	0.178	19.835	0.496	1.141	0.057	32.052	0.046	0.828	0.236	15.391	0.051
MR	0.043	0.043	22.692	0.567	1.370	0.069	126.184	0.180	0.760	0.217	28.044	0.093
LK-3	0.090	0.090	14.248	0.356	0.259	0.013	55.731	0.080	1.128	0.322	15.002	0.050
LK-4	0.044	0.044	14.112	0.353	0.456	0.023	66.805	0.095	1.223	0.349	12.694	0.042
LK-5	0.136	0.136	18.491	0.462	0.814	0.041	172.136	0.246	1.678	0.480	2.007	0.007
BL	0.446	0.446	17.532	0.438	3.403	0.170	489.572	0.699	3.064	0.876	83.032	0.277
TAT	0.347	0.347	15.098	0.377	0.788	0.039	246.268	0.352	3.030	0.866	67.466	0.225

Table S6: Values of estimated weekly intake (EWI, μg/kg body weight/day) and their percentages in comparison to provisional tolerable weekly intake (PTWI) for of 6 potential toxic metals in the total soft tissues of 21 populations of *Cerithidea obtusa* collected from the present study.

Sites	PTWI 361.5 Cd EWI	PTWI 217000 Cu EWI	PTWI 5642 Ni EWI	PTWI 347200 Fe EWI	PTWI 1302 Pb EWI	PTWI 434000 Zn EWI	%PTWI Cd EWI	%PTWI Cu EWI	%PTWI Ni EWI	%PTWI Fe EWI	%PTWI Pb EWI	%PTWI Zn EWI
SB-1	0.87	70.23	10.13	730.71	14.92	113.82	0.240	0.032	0.180	0.210	1.146	0.026
SB-2	2.81	107.96	4.80	185.08	4.47	95.96	0.778	0.050	0.085	0.053	0.343	0.022
KL-1	0.20	91.41	11.64	236.28	15.42	104.73	0.054	0.042	0.206	0.068	1.184	0.024
KL-2	1.32	111.92	4.19	257.44	11.19	108.64	0.364	0.052	0.074	0.074	0.859	0.025
KL-3	0.12	126.21	4.46	380.40	21.45	143.36	0.033	0.058	0.079	0.110	1.647	0.033
KL-4	0.13	136.90	7.17	328.66	29.96	102.05	0.035	0.063	0.127	0.095	2.301	0.024
KL-5	0.49	147.42	14.03	372.38	10.68	227.31	0.134	0.068	0.249	0.107	0.820	0.052
TK	4.78	124.28	13.64	233.70	17.50	132.09	1.322	0.057	0.242	0.067	1.344	0.030
KSA	7.63	100.93	14.03	400.90	36.63	135.72	2.111	0.047	0.249	0.115	2.814	0.031
SB-3	10.46	107.52	22.51	231.50	60.55	171.54	2.893	0.050	0.399	0.067	4.650	0.040
LK-1	2.52	51.59	17.67	364.46	6.56	100.72	0.696	0.024	0.313	0.105	0.504	0.023
LK-2	3.22	57.89	27.74	714.38	4.06	119.58	0.890	0.027	0.492	0.206	0.312	0.028
DR	1.65	120.93	12.19	522.97	21.43	117.63	0.457	0.056	0.216	0.151	1.646	0.027
BK	1.39	134.62	8.16	385.86	7.65	91.83	0.385	0.062	0.145	0.111	0.587	0.021
SM	1.24	138.84	7.99	224.36	5.79	107.74	0.344	0.064	0.142	0.065	0.445	0.025
MR	0.30	158.84	9.59	883.29	5.32	196.31	0.084	0.073	0.170	0.254	0.409	0.045
LK-3	0.63	99.74	1.81	390.12	7.90	105.01	0.175	0.046	0.032	0.112	0.607	0.024
LK-4	0.31	98.78	3.19	467.64	8.56	88.86	0.085	0.046	0.057	0.135	0.658	0.020
LK-5	0.95	129.44	5.70	1204.95	11.75	14.05	0.264	0.060	0.101	0.347	0.902	0.003
BL	3.12	122.73	23.82	3427.00	21.45	581.23	0.863	0.057	0.422	0.987	1.647	0.134
TAT	2.43	105.69	5.52	1723.88	21.21	472.26	0.672	0.049	0.098	0.497	1.629	0.109
